# Improved motor skills in autistic children after three weeks of neurologic music therapy via telehealth: a pilot study

**DOI:** 10.3389/fpsyg.2024.1355942

**Published:** 2024-05-08

**Authors:** Nicole Richard Williams, Corene Hurt-Thaut, Jessica Brian, Luc Tremblay, Marija Pranjić, Jessica Teich, Melissa Tan, Julia Kowaleski, Michael Thaut

**Affiliations:** ^1^Music and Health Science Research Collaboratory, Faculty of Music, University of Toronto, Toronto, ON, Canada; ^2^College of Music and Performing Arts, Belmont University, Nashville, TN, United States; ^3^Bloorview Research Institute, University of Toronto, Toronto, ON, Canada; ^4^Faculty of Kinesiology & Physical Education, University of Toronto, Toronto, ON, Canada; ^5^KITE Research Institute, University Health Network, Toronto, ON, Canada; ^6^Faculty of Medicine, Institute of Medical Science, University of Toronto, Toronto, ON, Canada

**Keywords:** autism, neurologic music therapy, motor, telehealth, sensory, BOT-2

## Abstract

**Background:**

Many autistic children experience motor skill deficits which can impact other areas of functioning, and research on therapeutic interventions for motor skills in autism is in a preliminary stage. Music-based therapies have been used extensively to address motor skills in non-autistic populations. Though a handful of studies exist on the effects of music-based therapies for movement in autistic children, none have investigated the possibility of administering sessions via telehealth. This mixed-methods pilot study investigated whether nine Neurologic Music Therapy (NMT)^®^ sessions via telehealth would improve motor and attention skills in autistic children.

**Methods:**

Five autistic children between five and 10 years of age participated in the study, with support from their caregivers. Motor skills were assessed using the Bruininks-Oseretsky Test of Motor Proficiency second edition, short form (BOT-2 SF), and a selective attention and sustained attention task were taken from the Test of Everyday Attention for Children, Second Edition (TEA-Ch2). Caregivers and the two neurologic music therapists involved in the study provided qualitative input about the perceived effectiveness of telehealth NMT for the children involved. Their responses were analyzed using qualitative content analysis. Caregivers also filled out a Sensory Profile 2 assessment prior to the onset of sessions so that each child’s sensory profile could be compared to their motor and attention results.

**Results:**

Statistically significant improvements in motor skills were observed between pre-test assessment and a two-week follow-up assessment. Results from attention test scores were not significant. Caregivers and neurologic music therapists generally perceived sessions positively and noted the importance of having caregivers actively involved. When compared with individual progress on the BOT-2 SF assessment, sensory profile results revealed that children with fewer sensory sensitivities tended to improve the most on motor skills. The improvements in motor skills and positive caregiver and therapist views of telehealth indicate that NMT motor interventions administered via telehealth are a promising avenue of therapeutic support for movement skill development in autistic children.

## 1 Introduction

Autism spectrum disorders (ASD, or autism) comprise a range of conditions involving difficulties with social communication and interaction as well as restricted or repetitive patterns of behaviors and interest (American Psychiatric Association, 2013). The prevalence of autism diagnoses has been increasing globally. The United States (U.S.) Centers for Disease Control and Prevention (CDC) reported that in the year 2000, around 1 in 150 children in the U.S. were diagnosed with the condition while with the most recent data from 2020, 1 in 36 eight-year-old children in the U.S. were diagnosed with autism (Centers for Disease Control and Prevention, 2023). In addition to the main diagnostic markers, autistic individuals often experience sensory hypo- and hyper-sensitivities, struggles with attention, and difficulties with motor skills (Fournier et al., 2010; Liu, 2013; LaGasse et al., 2019). Music-based interventions have increasingly been used to support neurodevelopmental skills in autistic individuals, including movement skills (Braun Janzen and Thaut, 2018). In recent years, clinicians and researchers have been exploring how to implement therapeutic interventions for autistic individuals via online video platforms as well as in-person (Solomon and Soares, 2020).

Though movement difficulties are not an official part of the primary autism diagnostic criteria, researchers have increasingly recognized what Kanner and Lesser (1958) observed, that autistic persons also display difficulties with motor functioning (Fournier et al., 2010; Bhat et al., 2011; Colombo-Dougovito and Block, 2019). In fact, technology that measures movement on a precise level can detect an autism diagnosis with extremely high reliability using movement differences alone (Torres et al., 2013; Milano et al., 2023). It is estimated that up to 90% of autistic children may experience motor difficulties such that they can receive a co-occurring diagnosis of developmental coordination disorder (Miller et al., 2021). Difficulties can be observed in gait and balance, arm motor functions such as reaching and grasping, speech motor functions, movement planning, and coordination (Fournier et al., 2010). Many of these motor difficulties in autism involve fundamental movement skills that are essential to child development and socialization: balance, locomotion, and object manipulation (Gandotra et al., 2020). Indeed, motor functioning is not just important to address for its own sake; poor motor skills are also associated with decreased outcomes in social, language, and cognitive areas like attention, memory, and executive functioning (Wilson et al., 2018; Zampella et al., 2021). High-quality intervention studies involving motor outcomes for autistic individuals are few though increasing [for reviews, see Colombo-Dougovito and Block (2019), Gandotra et al. (2020), Ruggeri et al. (2020), Frazão et al. (2023), and Ji et al. (2023)]. There is ample room for expansion of this research topic, particularly toward identifying replicable and generalizable interventions addressing motor skills for individuals on the spectrum.

The potential for music to be used as a motor intervention for autistic individuals is high (Hardy and LaGasse, 2013). There is substantial evidence for positive effects of standardized music-based interventions on motor impairments in conditions other than autism including cerebral vascular accident (stroke), Parkinson’s disease, traumatic brain injury, cerebral palsy, and more [reviewed in Braun Janzen et al. (2022)]. Music-based interventions are often successful in treating motor aspects of neurological conditions because the auditory system has extensive connections with motor areas in the brain such as premotor areas, basal ganglia, and the cerebellum (Grahn and Brett, 2007; Chen et al., 2008). Isochronous (and thus predictable) auditory cues entrain neurons of the auditory cortex, and prime motor areas to become ready to move [discussed in Braun Janzen et al. (2022)]. Engaging in active therapeutic music making has also been associated with improvements in neural connectivity and associated functional motor recovery across clinical populations (Sharda et al., 2018; Braun Janzen et al., 2022). Music-based therapies have been used to address many issues in autism such as social skill challenges, language and communication issues, and emotional/coping skills [see Braun Janzen and Thaut (2018), for a review]. This may be because autistic individuals often respond well to music, potentially due to increased sensitivity to musical parameters like pitch and a greater response in the inferior frontal gyrus (speech area) to sung versus spoken language (Kuhl et al., 2005; Lepistö et al., 2005; Sharda et al., 2015). Recent studies indicate that auditory-motor pathways appear to be functioning typically in individuals on the autism spectrum even though they often struggle with movement and sensorimotor integration (Tryfon et al., 2017; Edey et al., 2019; Jamey et al., 2019). Because autism is a highly heterogeneous condition, not every autistic person may respond well to musical stimuli (Ferrari and Harris, 1981; Ingersoll et al., 2003).

The research on music interventions used specifically for movement in autistic individuals is increasing. Srinivasan and Bhat (2013) reviewed a handful of studies investigating the effects of music-based interventions for motor difficulties in autistic persons, with many reports of positive results. More recently, Sharda et al. (2018) found that an 8–12-week music therapy intervention improved auditory motor connectivity in autistic children ages 6–12 years old. Srinivasan et al. (2015) found that autistic children engaged in a rhythmic-movement-imitation intervention (along with those in a robotics-movement group) improved on the body coordination composite of a motor assessment compared to a control group. Shemy and El-Sayed (2018) found significant improvements in bilateral coordination, balance, running speed and agility, and strength in 8–10-year-old children on the autism spectrum who received a three-month, three times-per-week Rhythmic Auditory Stimulation (RAS^®^) intervention compared to a control group who received physiotherapy. Imankhah et al. (2018) found that autistic boys who received 15 twice-weekly sessions involving music- and rhythm-based play and movement activities improved significantly more on motor coordination than those who did not receive treatment. Finally, a study by Shukla et al. (2022) sought to use traditional Indian Tabla drumming to promote upper-extremity motor skills needed for tooth-brushing. They reported basing their protocol on the recommendations of Thaut (1984) who promoted the use of carefully structured clinical music improvisation to address clinical goals with autistic children. Significant improvements were found in motor and social skills after the intervention in Shukla et al. (2022) study. These studies have promising results regarding the effects of music on movement in autistic individuals.

In addition to motor challenges, individuals on the autism spectrum are known to experience hypo- and hyper-sensitivities to sensory stimuli (American Psychiatric Association, 2013). Sensation (particularly in the visual and proprioceptive realms) is critical for motor functioning, and thus difficulties in sensory processing such as poor sensory integration or sensory sensitivities can influence motor difficulties in autistic individuals (Baranek, 2002, Liu, 2013; Muthusamy et al., 2021; Purpura Cerroni et al., 2022). Sensory sensitivities can thus affect autistic persons’ ability to engage in therapeutic or other activities. Thus, when developing interventions to address motor skills in autistic individuals, sensory sensitivities must be considered. Related to sensory issues is attention. Autistic individuals sometimes experience difficulty utilizing selective attention to focus on one aspect of incoming sensory information and inhibit others (LaGasse et al., 2019).

Research on interventions for sensory difficulties in autism is increasing [for reviews, see Case-Smith et al. (2015) and Weitlauf et al. (2017)]. Berger (2002) wrote a book on music therapy for sensory integration in autistic children, which provides a helpful conceptual overview on the topic based on her experience as a clinician along with anecdotal evidence. The book claimed that music engagement helps to anchor and organize autistic children’ sensory systems so that they can engage intentionally in their environments. Mertel (2014) outlined a protocol in which the NMT technique Auditory Perception Training (APT)^®^ can be used to facilitate sensory integration for populations including autistic individuals. In APT, individuals engage in interventions structured by an isochronous auditory beat along with multiple sensory inputs. By engaging in such interventions, sensory integration occurs, creating positive downstream effects on other areas of functioning such as cognition, executive functions, and execution of more complex movement skills. High-quality evidence for benefits of music-based interventions to address sensory difficulties in autism remains scarce. In their feasibility study, Lagasse et al. (2019) found that a music therapy attention intervention seemed to improve sensory gating in autistic children, though results were not statistically significant. The current study did not directly target sensory functioning aside from sensory-focused warm-ups at the beginning of sessions, as needed. The sensory profile of each child was considered when implementing motor interventions and interpreting results.

Online health services or *telehealth* was utilized in therapy with autistic persons prior to the 2019 Coronavirus Disease (COVID-19) pandemic, but its use increased dramatically since the pandemic began (Ellison et al., 2021). Telehealth has been used extensively with autistic individuals especially since the pandemic for diagnosis and therapeutic interventions [for reviews, see Stavropoulos et al. (2022) and Kane and DeBar (2023)]. Benefits of telehealth included: lower costs due to decreased travel time for therapists/clients (Lindgren et al., 2016; Kalvin et al., 2021; Su et al., 2021), increased parental engagement in therapy resulting in more transfer of skills to everyday life (Su et al., 2021), access for rural or remote clients (Ameis et al., 2020; Solomon and Soares, 2020; Simacek et al., 2021), and better engagement with the therapist online due to lower anxiety being in the comfort of their own homes (Kalvin et al., 2021). Disadvantages of telehealth therapy with autistic clients included: increased distractedness on computers or in the home environment (Kalvin et al., 2021), frustrations due to technical difficulties (Solomon and Soares, 2020; Su et al., 2021), and greater difficulty providing resources to parents (Solomon and Soares, 2020; Kalvin et al., 2021).

Prior to the pandemic, studies concerning the efficacy of online music therapy for autistic clients were limited to a single case study about an autistic teen by Baker and Krout (2009). The teen had previously engaged in in-person music therapy, later switching to music therapy via telehealth. Baker and Krout (2009) reported that telehealth music therapy was more effective in promoting self-expression and emotional engagement in therapy than in-person therapy. Williams et al. (2024) reported that a music intervention for language goals implemented via telehealth yielded higher engagement in autistic children than a non-music telehealth intervention for language goals. Liu et al. (2023) reported that parents perceived their autistic children broadly improved in social and play skills after a 10 weeks of hour-long Music Enhanced Reciprocal Imitation Training sessions. In previous work by Richard Williams et al. (2022), qualitative survey data from music therapists working with autistic children over telehealth indicated that telehealth music therapy was possible and music therapists continued to address clinical goal areas for autistic clients, given sufficient technological resources and caregiver support. Attention skills were reported as another important mediating factor associated with the ability to engage in telehealth (Richard Williams et al., 2022). Given the importance of attention for sensory regulation and engagement in telehealth, it was important to assess attention skills as part of the current study.

Research on motor interventions implemented over telehealth for autistic people is limited to one study with preliminary results by Cleffi et al. (2022). In their report, Cleffi et al. (2022) described an ongoing randomized control trial that they translated from in-person to telehealth. They worked with autistic children and their caregivers over Zoom (Zoom Video Communications Inc., 2016), providing deliveries of materials to each family, and guiding them through various games and play-based interventions that addressed motor skills. Cleffi et al. (2022) described that movement interventions implemented with family assistance appeared successful over telehealth, and pre- and post-testing using the Bruininks-Oseretsky Test of Motor Proficiency, 2nd Edition (BOT-2) (Bruininks and Bruininks, 2005) and Test of Gross Motor Development (TGMD) will reveal whether there is a difference in results between telehealth motor groups and a parallel in-person motor intervention group. There are currently no published studies examining the effects of music-based interventions on movement in autistic children over telehealth.

The current study piloted the implementation of Neurologic Music Therapy (NMT)^®^ interventions (rhythmic auditory stimulation [RAS^®^], patterned sensory enhancement [PSE^®^], and therapeutic instrumental music performance [TIMP^®^]) via telehealth in collaboration with caregivers to address motor functioning in autistic children. NMT is an evidence-based set of music-based interventions grounded in research of music perception and cognition (Thaut and Hoemberg, 2014). The three techniques used in the current study, RAS, PSE, and TIMP are motor techniques that have been researched extensively in other clinical populations (Braun Janzen et al., 2022), but very little with autistic persons (Shemy and El-Sayed, 2018), and never directly researched in an intervention study over telehealth (Cole et al., 2021). Music-based therapists practicing NMT lost significantly fewer clinical hours than music-based therapists practicing other models of music therapy, indicating that NMT interventions may be particularly transferable to telehealth (Richard Williams et al., 2024).

The current pilot study was designed to investigate: (1) Do NMT motor techniques (RAS, TIMP, PSE) applied via telehealth improve (a) motor skills and (b) attention in autistic children? (2) What did caregivers and parents perceive as the positive and challenging aspects of the sessions? (3) Did the degree of sensory challenges affect children’s ability to participate in and benefit from telehealth NMT?

## 2 Materials and methods

### 2.1 Participants

Five autistic children aged five to 10 years old (four male, one female) and their caregivers were recruited from a large organization serving a diverse population in the Greater Toronto Area. All parents signed a consent form on behalf of their children prior to participating in the study, and each child also signed an assent form which explained the study in a simplified manner. See Table 1 for demographic information. The study also involved four neurologic music therapists: one as the assessor, two who ran sessions (from hereon “therapists”), and one other who helped with qualitative content analysis and acted as a second assessor for one participant to assess inter-assessor reliability. Neurologic music therapists are certified music therapists who have taken additional training in NMT theory and techniques from the Academy of Neurologic Music Therapy^®^. The therapists who carried out the assessment sessions and intervention sessions had experience working with autistic clients.

**Table 1:** Participant demographics.

Characteristic	*n*	*%*
Gender		
Male	4	80
Female	1	20
Race/Nationality (self-described)		
Sri Lankan – Canadian	1	20
Indian – Canadian	1	20
Caucasian (Armenian)	1	20
Asian/Indian	1	20
Asian American	1	20

Participant age range was 5–10 years.

### 2.2 Methodology

This pilot study employed a mixed-methods approach. Mixed-methods research is employed when neither qualitative nor quantitative data alone are sufficient to adequately address a problem, and when more insight can be gained from a combination of both qualitative and quantitative approaches (Creswell, 2015). Telehealth music therapy is a fairly new approach, so gathering qualitative in addition to quantitative data in the current study helped to provide rich information about whether telehealth music therapy was an effective and feasible method for addressing motor skills in autistic children with the support of their caregivers. Quantitative data (including descriptive data) helped to provide a more objective measure of whether telehealth music therapy was effective in addressing specific goal areas.

The current study’s design utilized a version of an explanatory sequence method within an intervention mixed methods design (Creswell, 2015). In an explanatory sequence model, quantitative data are collected and analyzed before and after a clinical intervention is applied, and qualitative data are collected and analyzed at the end of the study to help explain or interpret the quantitative data. Because all interview forms and assessment instruments had to be submitted during the research ethics approval phase, qualitative interview forms were created at the outset of the study, and quantitative and qualitative data were integrated during the final, interpretive stage of data analysis. This study received ethics approval from the University of Toronto Research Ethics Board.

#### 2.2.1 Philosophical approach

The first author’s philosophical approach for the current study is pragmatic. A pragmatic study identifies a specific, practical problem, and often uses mixed methods to better understand and address the problem from multiple viewpoints (Creswell and Poth, 2018). The current study identified the problem as: Can NMT motor interventions be implemented effectively online with autistic children who are supported during sessions by their caregivers?

### 2.3 Intervention sessions and materials

#### 2.3.1 Materials and overall structure

Each family was loaned a bin of instruments and assessment materials for the duration of the training and assessment period. Sessions included one pre-intervention assessment, nine 45-min music therapy sessions spread over three weeks (three sessions per week), a post-assessment, and a follow-up assessment session that took place two weeks after the post-assessment session (12 sessions in total, including assessment and intervention sessions). The instrument/assessment kit was picked up after the final assessment, sanitized, and then used for subsequent participants. All intervention and assessment sessions were led by therapists over Zoom (Zoom Video Communications Inc., 2016). Caregivers participated in all sessions with their child and helped to facilitate some aspects of interventions led by the therapist.

#### 2.3.2 The intervention

Intervention sessions were largely comprised of NMT interventions tailored to address motor skills assessed on the BOT-2 SF such as fine motor precision and integration, manual dexterity, bilateral coordination, balance, ambulation, upper-limb coordination, and strength. Three NMT motor interventions were used: TIMP, which involves engaging the participant in playing musical instruments to practice certain movements, for example tapping a castanet to practice finger dexterity; PSE, which involves a therapist providing accompaniment that supports and drives movement, for example using rhythmic music with an ascending and descending melody to support pressing arms up and controlling a downward motion during push-ups; and RAS, which is the use of a metronome to assist with repetitive rhythmic movements such as gait. Participants who presented with signs of sensory-seeking behaviors that made it difficult for them to engage in the motor interventions right away received a brief sensory input intervention. The therapist would direct the participant’s caregiver to deliver squeezes or pats to the child’s body (feet, calves, quads, hips, head, back/chest, shoulders, arms, and hands/fingers), spending 1–2 min for each body part. A rhythmic song with directive lyrics and metronome helped to guide the sensory exercise. All intervention sessions were video-recorded with written permission of participants. See Supplementary Table S1 for the description of intervention protocols.

### 2.4 Assessment

#### 2.4.1 Timeline of assessments

Prior to the motor and attention assessments, caregivers filled out an intake form collecting demographic data and information on musical preferences. Assessment tools included in the pre-test, post-test, and two-week follow-up included the short form of the BOT-2 (BOT-2 SF), and a selective attention and sustained attention task taken from the Test of Everyday Attention for Children, Second Edition (TEA-Ch2) (Bruininks and Bruininks, 2005; Manly et al., 2016). Assessment tools included in pre-test only were the SP2 (Dunn, 2014) and an intake form collecting demographic data and information on musical preferences. A qualitative questionnaire regarding the caregiver experience, and a qualitative questionnaire regarding neurologic music therapist experience were administered after the final assessment session (two-week follow-up). Finally, after each session, therapists would fill out a checklist to report on the amount of time the child spent fully engaged during each session and report any parent questions or any deviations from protocol. A copy of this checklist can be found in Supplementary Material S2. Informal conversations between the researcher, assessor, and therapists regarding the feasibility of aspects of the study were recorded and comprise additional qualitative data.

#### 2.4.2 Implementation of assessments

BOT-2 SF and TEA-Ch2 assessments were implemented on video by a trained assessor over according to directions from the publisher Pearson on virtual assessment implementation. Testing objects were loaned to families along with the instrument kit, and caregivers helped to set up materials for assessments according to directions from assessors. Assessment elements that could be scored live utilized live scoring by the assessor, and other portions involving paper were scored once the box of musical instruments and assessment resources were returned after the study was complete. Assessment sessions were not recorded except for P5’s assessment sessions, which were additionally scored by a second assessor to evaluate consistency of assessment.

#### 2.4.3 Instruments

The BOT-2 (Bruininks and Bruininks, 2005) it is one of the most reliable assessments used to assess progress in motor skills in motor-intervention studies for autistic children (Dietz et al., 2007; Wilson et al., 2018; Downs et al., 2020; Ruggeri et al., 2020). It has been used to measure motor outcomes for autistic children via telehealth (Cleffi et al., 2022). The longer BOT-2 assesses motor functioning in four sub-areas: fine motor control, manual coordination, body coordination (balance, posture), and strength/agility. The short form provides a measure of general motor functioning amalgamated across the four sub-areas from the larger form. Sample tasks on the short form include tracing different shapes, sorting pennies, bouncing a ball between two hands, standing balance exercises, and sit-ups. Each task is scored, and total points calculated as a single number, which is then scaled according to the child’s age and sex. Though the BOT-2 SF test is reported to have a high degree of reliability (Downs et al., 2020), we had a second assessor independently score assessment videos recorded for one of the participants to double-check the reliability of the primary assessor’s work. The two assessments for the participant were within one scaled point of one another and had identical slopes between the three time-points.

The TEA-Ch2 (Manly et al., 2016) is a collection of tasks designed to assess different types of attention: selective, sustained, divided, and alternating. Other NMT intervention studies have used the TEA-Ch2 to assess progress in attention in autistic children as a result of NMT attention interventions (LaGasse et al., 2019; Sa, 2020). The current study was not implementing attention interventions, but because engaging in the motor interventions required attention, and participants were required to sustain their attention during each 45-min session (although most took breaks), we wanted to measure if there were secondary effects on selective and sustained attention. Thus, subtests from the TEA-Ch2 measuring selective and sustained attention were included in the study: the Hector Line Cancelation Test (selective attention, paper test involving crossing off specific lines) and Sustained Attention to Response Task (SART, computer test). The selective attention (line crossing) task was included in paper booklets given to families in the instrument kit dropped off at their homes. The SART task involved watching a series of shapes appear on a computer screen and tapping a key in response to each shape except one specific shape.

The Sensory Profile 2 (SP2) is questionnaire given to parents regarding their child’s level of sensory responsiveness (Dunn, 2014) and is one of the most tools for assessing and discussing sensory sensitivity for autistic individuals (He et al., 2023). The SP2 includes various booklets for appropriate for various age categories, and is grounded in neuroscientific understanding of how children respond to sensory stimuli in their environment (Dunn, 2014). The SP2 aims to identify the child’s neurological sensory threshold and pattern of behavioral self-regulation in seven areas of sensory processing: general, auditory, visual, somatosensory (touch), vestibular (movement/balance), proprioceptive (body position), oral sensory, and overall sensory processing. Thus, the questionnaire helps to place the child in one of the four quadrants of the Dunn (2014) SP2 diagram for each sensory area. The SP2 questionnaire booklet appropriate for each participant’s age was included in the box of instruments. Parents were instructed to fill it out before intervention sessions began and kept it in the box of instruments to be returned and scored by the lead researcher once sessions were complete.

Questionnaires were given to the therapists and each participant’s caregiver after the final (follow-up) assessment session. For each participant with whom they worked, therapists were asked: (1) What was the most positive aspect of facilitating sessions/assessments? (2) What was the most challenging aspect of facilitating sessions/assessments? (3) Is there anything that could be helpful for other neurologic music therapists facilitating sessions/assessments via Zoom? (4) Is there anything else you would like to say about your experience as therapists in this study?

Each caregiver was asked: (1) What was the most beneficial aspect of the study for your child and for you? (2) What was the most challenging aspect of this study for you and your child? How would you rate your experience of online Zoom sessions, from a scale of 0 (not beneficial) to 10 (extremely beneficial). (3) Is there anything else you would like to say about your experience with the study? (4) If it was possible, would you be interested in registering your child for online or in-person NMT sessions?

### 2.5 Data analysis

#### 2.5.1 Motor and attention outcomes

Due to difficulties the clients had performing the sustained attention assessment, attention data collected from the SART was determined not meaningful and was not analyzed. The assessor reported that the SART was extremely difficult to administer over telehealth.

Aggregate data from the BOT-2 test and selective attention (line-crossing) tasks were analyzed using one-way repeated-measures ANOVAs in the data analysis software R (R Core Team, 2022). Tests of normality and homogeneity of variance were performed on the motor and selective attention data sets. Mauchly’s test of Sphericity was calculated as part of the analysis, and if needed, Greenhouse-Geisser corrections were automatically applied to any factors violating this assumption.

In the motor data, there were no extreme outliers, and the Shapiro–Wilk test indicated that the data was normally distributed (all *p-*values were > 0.05). In the selective attention data, there was one extreme outlier in the first time point, and the data in the first time-point violated the Shapiro–Wilk test of normality (*p* = 0.04).

#### 2.5.2 Qualitative analysis

A qualitative content analysis (QCA) was performed on the answers to questions in the assessments to search for and identify common themes. QCA assesses data in domains that are not yet well-understood, particularly in healthcare (Hsieh and Shannon, 2005; Elo and Kyngäs, 2008). Two individuals performed the QCA: the first author and another PhD candidate who was not one of the therapists or main assessors. Both individuals read the responses to questions, and independently identified and categorized responses according to common themes in an electronic codebook. Software was not used in the qualitative analysis. When interpreting and categorizing participants’ contributions, both individuals strived to maintain awareness of biases and opinions which could influence this process by writing down thoughts in the margins of the codebook as they pertained to the emerging themes (Creswell, 2015). After independently coding responses, the first author compared both codebooks and compiled themes into a single document. The two individuals discussed the themes and finalized which categories seemed to be the most salient. The two therapists whose data were assessed are colleagues of the two individuals assessing the data. No relationships between either of the individuals performing the QCA and the caregivers existed beyond contact made by the author and the caregivers during the recruiting process. Member checking was employed with therapists, but not participants’ caregivers.

#### 2.5.3 Sensory profile comparison

Motor results, qualitative responses, and data from the SP2 (Dunn, 2014) were compared alongside one another to illuminate possible trends or connections between sensory sensitivities on the SP2 and ability to engage in the telehealth intervention sessions.

## 3 Results

### 3.1 Motor outcomes

**Figure 1:**
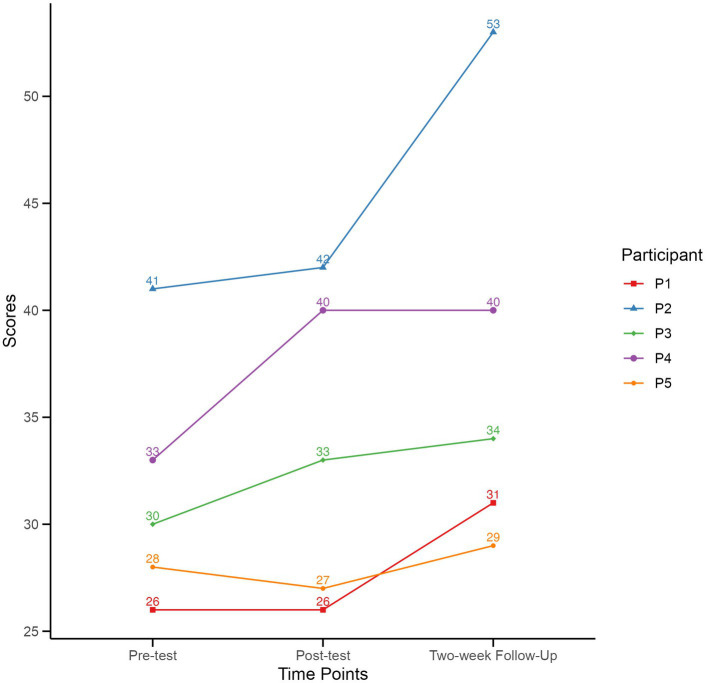
Individual scaled BOT-2 scores across the three assessment time-points.

A one-way repeated-measures ANOVA was conducted on the scaled scores of the BOT-2 SF assessment results to ascertain the effects of the intervention on participants’ motor performance over time (pre, post, and two-week follow-up). The ANOVA was performed using R (R Core Team, 2022). There was a statistically significant difference between average scores for at least two time points *p* = 0.03. A Tukey *Post Hoc* test could not identify at α *=* 0.05 significance level the exact location the difference, which trended to be between the pre-test and two-week follow-up test (*p* = 0.23). Visual inspection of a graph of the BOT-2 SF scaled scores corroborate that the scores increased between the pre- and follow-up test. See Table 2 and Figure 1.

**Table 2:** Means, standard deviations, and one-way analyses of variance in BOT-2 SF and selective attention (line crossing) scores.

Measure	Pre-test		Post-Test		Follow-Up		*F*(2, 8)	η^2^_G_
	*M*	*SD*	*M*	*SD*	*M*	*SD*		
BOT-2 SF	31.6	5.9	33.6	7.3	37.4	9.7	5.612^*^	0.107
Line crossing	5.2	5.1	5.0	5.1	5.4	4.0	0.085	0.001

^*^p < 0.05.

### 3.2 Attention outcomes

The TEA-Ch2 Line Crossing Task was completed by most participants independently. Two participants struggled with the task on certain trials, and caregivers either helped them or simply allowed them to perform the task incorrectly (e.g., connecting lines rather than crossing them out). The one-way repeated measures ANOVA was not statistically significant (*p* = 0.92). See Table 2.

### 3.3 Qualitative outcomes

The QCA found three major themes across both the caregiver and therapist responses: (1) Caregiver involvement was necessary and beneficial, (2) clients benefited from sessions, and (3) engagement was sometimes limited due to distractions. The three major themes along with constituent categories are represented in Supplementary Table S3. Quotes from caregivers are marked with a “C” while quotes from the therapists are marked with a “T.” In general, caregivers as well as therapists held a positive view of the music therapy sessions. Caregivers often remarked that their child engaged well over Zoom for music therapy in a way that they did not for other (non-music-based) therapies. Therapists also perceived participant skill improvements during music therapy sessions. The limits of virtual sessions were acknowledged, since distractions and sensory needs made it difficult for participants to engage at times. Caregivers articulated benefits of being involved in sessions themselves, and therapists similarly articulated that sessions would not be possible without caregiver support and involvement. Please refer to Supplementary Table S3 for a delineation of therapist and caregiver responses.

### 3.4 Sensory outcomes and comparison

Results from each client’s SP2 (Dunn, 2014) assessment given prior to the intervention period was compared alongside individual quantitative results. See Table 3. Patterns emerged, although with the small sample generalization is not possible. Those with the three highest percent-change in motor scores also had each four or fewer areas of sensory sensitivity and were reported to have consistent engagement. The two children with the most sensory sensitivities showed the lowest percent-change improvements in their BOT-2 scores.

**Table 3:** Comparison of participant sensory factors, age, and engagement.

ID	Age	Percent-change in BOT-2 SF score from pre-test to follow-up	Parent rating of sessions	# of sensory areas 1 SD outside normal range	# of sensory areas 2 SD outside normal range
P1	9	19.2	8	3/19	0/19
P2	9	29.3	N/A	4/19	0/19
P3	5	13.3	5	4/19	6/19
P4	6	21.2	10	1/19	0/19
P5	10	3.6	10	7/19	3/19

The scale for parent rating of sessions is as follows: (10 = extremely beneficial and 0 = not beneficial).

Participants with greater sensory struggles, particularly if they were younger, perhaps would have benefited from more direct intervention to address sensory issues alone prior to engaging in intensive sessions addressing motor skills.

## 4 Discussion

### 4.1 Motor improvements

The participants showed statistically significant increases in motor skill performance measured by the BOT-2 SF test. Visual inspection of data revealed that motor assessment scores on the final (follow-up) assessment were higher than those on the initial test. This result implies that motor skills continued to improve in the two weeks after the final intervention. One possible explanation for this pattern of results is that that offline gains may have occurred between the assessment that occurred soon after the last session and the two-week follow up, allowing for motor skills to solidify and be observed on the final follow-up assessment. The term “offline gains” refers to improvements in motor skill that happen following an interval of time in which motor skills previously practiced are consolidated, but not actively practiced (Lugassy et al., 2018). Motor consolidation occurs when sleep and rest occur after intentional motor practice, as first observed by Brashers-Krug et al. (1996). The present study intentionally spaced sessions at least 48 hours apart to allow for motor consolidation between training sessions. Future studies could explore the effects of music-motor interventions on functional connectivity in autistic individuals, along with behavioral motor assessment measures. Previous studies have set a promising precedent for such research: Sharda et al. (2018) found that engaging in 8–12 sessions of music therapy targeting social interaction increased functional connectivity between auditory and motor areas in autistic children, relative to those in a control group. Their study also saw a decrease in over-connectivity between auditory and visual-association areas. There is theoretical support for improvement based on music-based interventions targeting motor skills also. D’Mello and Stoodley (2015) reported that autistic individuals show overconnectivity between the cerebellum and motor cortices, which is associated with underconnectivity in cerebro-cerebellar pathways for language and social interaction. Braun Janzen and Thaut (2018) further theorized that music-based motor engagement could help to improve cerebro-cerebellar connectivity, given that music and rhythm activates the cerebellum along with motor areas. Future research can investigate the relationship between scores on a motor assessment and neural correlates such as functional connectivity between cerebellar and cortical brain regions.

Another possible explanation for the increased scores after the two-week follow-up period is that parents may have begun to practice motor skills with their children even after the therapy period was complete. Though no parents directly shared that they were practicing the motor skills after sessions were complete, some parents did report gaining new skills to support their child, so this possibility cannot be ruled out.

Although the current study contains many of the limiting factors described in Srinivasan and Bhat (2013) such as a small sample size and no control group which limit generalizability, the NMT interventions used in this study (TIMP, PSE, and RAS) are specific and replicable. Results indicate that follow-up research can be conducted using these consistent NMT intervention protocols to investigate replications of the current outcomes.

The promising motor results echo those of Srinivasan et al. (2015), Imankhah et al.’s (2018), Shemy and El-Sayed (2018), and Shukla et al. (2022), who all found that music-based interventions improved movement skills in autistic participants. In particular, Imankhah et al. (2018) used exercises which resemble the techniques used in the current study such as TIMP and RAS. The current study adds to previous data by providing evidence that it may be possible to address motor skills in autistic children via telehealth, and supports the development of larger studies to investigate the benefits of NMT motor interventions for children on the autism spectrum.

### 4.2 Inconclusive attention data

Due to many participants being unable to complete the SART assessment independently, attention outcomes for the sustained attention were inconclusive. Analyses for the selective attention assessment were not statistically significant. These results imply that, first, the SART attention task was either too advanced for the children taking the tests, too difficult to administer via telehealth, or both. Second, the lack of even a trend toward improvement in the selective attention task indicates that selective attention did not improve over the course of the study, which perhaps should not be surprising given that the interventions in the study were not targeting attention skills. Though studies by Pasiali et al. (2014), Lagasse et al. (2019), and Sa (2020) found that NMT improved attention skills (measured by the TEA-Ch2) in autistic adolescents, the subjects in that study received attention-specific interventions and were older, so better able to carry out the assessments. It is not possible to make any firm conclusions related to attention in the current study.

### 4.3 Positive qualitative responses

Caregivers and therapists expressed an overall positive view of the telehealth sessions, despite the presence of occasional challenges. This result is in keeping with prior research indicating that the opportunity to access services online is seen positively (Cole et al., 2021; White et al., 2021). None of the families in the study had previously accessed music therapy, and several of the families found sessions beneficial enough that they requested information about how to find NMT services for their child after the study was complete. None of the caregivers specifically mentioned (nor were they directly asked) whether the number of sessions (nine, over 3 weeks) felt feasible for them, but noteably each of the five participants and their caregivers attended each one of their assessment and training sessions, with only one participant ending a session early one time. This 100% study participation rate indicates that implementing NMT motor interventions over telehealth is not only likely effective for motor development, but feasible for families. Anecdotal comments from some parents (outside the qualitative questionnaires) indicated that they perceived their children to be benefitting tremendously from the sessions and were learning new ways to support their children because of the sessions.

### 4.4 Sensory implications

Like results found in White et al. (2021) and Richard Williams et al. (2022), participants with fewer sensory sensitivities tended to engage more consistently over telehealth and made more progress in motor skills than their peers with greater sensory sensitivities. This result resonates with recent research that found autistic children with sensory sensitivities tend to struggle with attention (Dellapiazza et al., 2018), and that challenging behaviors in autistic children (including inattention) can be explained to a high degree by the presence of sensory sensitivities (Dellapiazza et al., 2020). Thus, participants with more sensory issues may have struggled to maintain attention and behave in ways conducive to engagement over telehealth during sessions more than others who had fewer sensory difficulties.

Therapists indicated on the fidelity checklists (Supplementary Material S2) that all participants spent the goal minimum of 75% of session time doing NMT motor interventions. Therapists sometimes provided a proprioceptive-input intervention at the beginning of sessions, but this intervention may not have been sufficient in terms of length and the fact that only one sensory area (proprioception) was addressed. It is possible that children with higher sensory needs could benefit from full sessions directly addressing these sensory needs as a prerequisite to working on movement skills directly. Future studies should investigate the impact of degree and type of sensory sensitivities and age on ability to engage in telehealth music therapy. Research should also investigate the efficacy and feasibility for NMT interventions for sensory needs in autistic children.

### 4.5 Limitations

The small sample and lack of control group limit the generalizability of these outcomes but the study results provide a promising and replicable context for future investigations. In terms of the motor assessment results, the pattern of improvement from pre-test to follow-up test only occurred clearly for two participants, whereas the others there was more of a plateau after the post-test. Along these lines, it is possible that one participant (participant two) may have been driving the change. Replicating this study with a much larger sample would help to identify if these results are meaningful and generalizable. The impact of sensory challenges on motor skills must be interpreted with caution as it was underpowered for formal analysis. In addition, because the presence of other autism symptoms was not directly measured, it is possible that participants with greater sensory challenges also had more intense autism symptoms in general which impacted their ability to benefit from the intervention. There are several other limitations that should be considered to improve upon this pilot study in the future. The fact that therapists sometimes began sessions with a brief sensory intervention may have introduced a confound, as it is not possible to know if gains in the sessions could be due to the motor interventions or also in part to the sensory interventions. In terms of assessment, because the same assessor was present at all three time points, it is possible that assessor bias was introduced that influenced the interpretation of motor scores as improving over time. The addition of a second assessor for one of the participants, who was blinded to the time of assessment and found a similar pattern of results, helps to mitigate the possibility of bias only partially. Finally, the attention tasks were largely too difficult for children to do, and difficult to implement via Zoom.

## 5 Conclusion

This small pilot study found improvements in motor skills in autistic children following nine sessions of motor-based interventions delivered by neurologic music therapists. Caregivers and therapists felt that the children improved during NMT sessions, and caregivers felt that they learned new strategies for helping their children during sessions. Observations of sensory sensitivities combined with individual session progress indicated that participants with fewer sensory sensitivities, or who were older, tended to engage the most consistently over telehealth and improve the most in motor skills. The results from this pilot study support the initiation of future research with larger samples and a control group in ascertaining how NMT motor interventions can benefit autistic children both in-person and via telehealth.

## Data availability statement

The datasets presented in this article are not readily available because of the small sample. Requests to access the datasets should be directed to nicole.richardwilliams@belmont.edu.

## Ethics statement

The studies involving humans were approved by University of Toronto Research Ethics Board. The studies were conducted in accordance with the local legislation and institutional requirements. Written informed consent for participation in this study was provided by the participants’ legal guardians/next of kin.

## Author contributions

NR: Conceptualization, Formal analysis, Investigation, Methodology, Project administration, Resources, Visualization, Writing – original draft, Writing – review & editing. CH-T: Supervision, Writing – review & editing. JB: Supervision, Writing – review & editing. LT: Supervision, Writing – review & editing. MP: Data curation, Writing – review & editing. JT: Data curation, Project administration, Writing – review & editing. MTa: Data curation, Writing – review & editing. JK: Formal analysis, Writing – review & editing. MTh: Supervision, Writing – review & editing.
