# Supplementary material for: Improved motor skills in autistic children after three weeks of neurologic music therapy via telehealth: a pilot study
Source: Front Psychol. 2024 May 8;15:1355942. doi: 10.3389/fpsyg.2024.1355942 (PMC11110893; doi:10.3389/fpsyg.2024.1355942)
Supplement: Supplementary file 1 [file Table_1.pdf]

frontiers

Supplementary Material

Improved motor skills in autistic children after three weeks of neurologic music therapy via telehealth: A pilot study

Nicole Richard Williams[1,2*], Corene Hurt-Thaut[1], Jessica Brian[3], Luc Tremblay[4,5], Marija Pranjić[1], Jessica Teich[1], Melissa Tan[1], Julia Kowaleski[1], Michael Thaut[1]

[1]Music and Health Science Research Collaboratory, University of Toronto, Faculty of Music, Toronto, ON, Canada

[2]Belmont University, College of Music and Performing Arts, Nashville, TN, United States of America

[3]Bloorview Research Institute, University of Toronto, Toronto, ON, Canada

[4]Faculty of Kinesiology and Physical Education, University of Toronto, Toronto, ON, Canada

[5]KITE Research Institute, University Health Network, Toronto, ON, Canada

*** Correspondence:**
Nicole Richard Williams: Nicole.richardwilliams@belmont.edu

1 Supplementary Material 1

Neurologic Music Therapy (NMT) Intervention Protocols

Part 1: Training Session Protocol

Session 1:

A. Therapist starts session by building rapport with child and parent, potentially starting with an engaging familiar song.
B. Therapist demonstrates target movement, and/or instructs parent to demonstrate movement for their child.
C. Therapist uses a tap metronome to determine tempo selected by participant and records tempo of exercise.
D. Therapist uses live or recorded music and a metronome rhythm to structure the exercise according to the relevant neurologic music therapy technique.
E. Repeat B-D for remaining exercises.

Sessions 2 - 9:

A. If needed, therapist starts session by building rapport with child and parent, potentially starting with an engaging familiar song.
B. Using the tempo previously selected by the participant for the exercises, the therapist demonstrates the task and/or invites the parent to demonstrate. The participant is then invited to play at the same
C. tempo with the metronome, while the therapist makes any necessary adjustments to instrument positioning.
D. The therapist provides rhythmic musical accompaniment, while continuing to monitor the participant closely. Complexity of the task may be increased; e.g. changing positioning of instruments, altering tempo.
E. Repeat B-D for remaining exercises.

Part 2: Detailed Description of NMT Interventions

Song ideas are provided to therapists, who will adapt and tweak songs to be appropriate for each participant's musical preferences and age. Each musical exercise is based on the corresponding exercise from the BOT-2 motor assessment. See the following table:

BOT-2 Exercise(s)	NMT Intervention (TIMP, PSE, RAS)	Target Speed/Reps
Fine Motor Precision: Drawing Lines through Paths—Crooked AND Fine Motor Integration: Copying a Square	Basic Concept:<br>Child traces shapes in a stencil.<br>Song idea:<br>Verse: "What kind of sound does a circle (or other shape) make? What kind of song does a circle make? Let's hear you play a circle, let's hear you play a circle."<br><br>Chorus: Child draws circle (or whatever shape) inside the stencil, and therapist uses PSE to create a specific accompaniment pattern to drive/support the movement.<br><br>Variation: Once the child knows the sounds corresponding to each shapes, therapist could play a game where the child must respond to whatever musical sound the therapist plays to draw the corresponding shape.<br>NMT Technique: PSE	Accuracy matters only – speed/timing not as crucial. The fewer errors the better.
Fine Motor Precision: Folding Paper AND Manual Dexterity: Transferring Pennies	Basic Concept:<br>Child holds and uses a pick to strum against washboard; uses varying thicknesses of pick, becoming thinner and thinner.<br>Song idea:<br>Therapist can use any song that can be played with varying volumes and speeds. Louder volumes would correspond to using a thicker pick on the washboard.<br>NMT Technique: TIMP	Accuracy, ability to hold the pick matters.
Fine Motor Integration: Copying a Square AND Fine Motor Integration: Copying a Star	Basic Concept:<br>Place finger-bells in a star or square shape (star: one bell for each point; one bell for each indent; square: four bells). Child can play random bells during a verse, then during a chorus, must play the bells in the star shape.<br>NMT Technique: TIMP	Accuracy, aiming for playing each bell in order.

BOT-2 Exercise(s)	NMT Intervention (TIMP, PSE, RAS)	Target Speed/Reps
Manual Dexterity: Transferring Pennies	Basic Concept:<br>Passing game: caregiver puts shaker egg in front of child's preferred hand. Child picks up shaker egg, transfers to other hand, and drops into a drum or other container. Caregiver can continue taking shaker eggs from container to pass to child in a circular manner.<br>To increase difficulty: Speed up the chant gradually; Change from shaker eggs to picks (which more resemble pennies as in the BOT-2 assessment).<br>Song Idea:<br>Chant-like song to help structure the intervention. Like: "Catch the egg, change hands, drop it, get ready!" Caregiver must be ready to hand the child a shaker egg each time.<br>Use a metronome to structure intervention.<br>NMT Technique: TIMP; or RAS (if passing item that does not create sound).	# of shaker eggs passed in 15 seconds (max goal of 20 shaker eggs)
Bilateral Coordination: Jumping in Place—Same Sides Synchronized	Basic Concept:<br>Caregiver holds a drum up for child alternating between holding it on the right and left side (e.g. a shape drum that can be played on both sides). Child has hand and foot tambourines on each foot (optional). Essentially, this intervention involves doing rhythmic jumping back and forth like in the BOT-2 task, but with hand drumming & foot tambourines.<br>Song Idea:<br>For the chorus, the caregiver is facing the child, and holding two drums.<br>Verse: Child-selected action (e.g., clapping hands, etc.). Child and caregiver can do the action together.<br>Chorus: Get into position, put right foot forward, right arm forward on the drum, left hand back, and jump. (Caregiver is getting drums ready at this part for child to tap).<br>NMT Technique: TIMP	5/5 jumps with correct form
Bilateral Coordination: Tapping Feet and Fingers—Same Sides Synchronized	Basic Concept:<br>Two drums on table, *or* in caregivers' hand, one on right and one on left side. Child is wearing foot tambourines on toes. Child alternates tapping right foot and tapping right hand on drum, and doing so with left hand and foot.<br>Song Idea:<br>As in other bilateral coordination activity.<br>NMT Technique: TIMP	10/10 taps without pauses or errors.

BOT-2 Exercise(s)	NMT Intervention (TIMP, PSE, RAS)	Target Speed/Reps
Balance: Walking Forward on a Line	Basic Concept:<br>Child walks on a line on the floor made of tape. Family can place a piece of tape on floor for child to walk on.<br>Song Idea:<br>Song about walking on a tightrope, where the child does some "get ready" movements like: take a deep breath, stretch your arms up and down, shake out the jitters, and ready to walk! Then would commence walking heel-toe, heel-toe, etc.<br>NMT Technique: RAS pre-gait, (PSE for heel-toe walk).	6 sequential steps in a line.
Balance: Standing on One Leg on a Balance Beam—Eyes Open	Basic Concept:<br>Child stands on one leg on piece of tape on floor for up to ten seconds.<br>Song Idea:<br>Verse: Various fun movements (stomping feet, clapping hands, etc).<br>Chorus: Child stands on leg with hands on hips and must make it through chorus while standing on one leg.<br>NMT Technique: PSE	Test duration of standing on one leg – 10 seconds.
Running Speed and Agility: One Legged Stationary Hop	Basic Concept:<br>Child hops on one leg for 15 seconds.<br>Song Idea:<br>Verse: Various fun movements (stomping feet, clapping hands, etc).<br>Chorus: Child must hop continuously on one foot during duration of chorus (15 seconds)<br>NMT Technique: PSE	Test duration of jumping on one leg – 15 seconds.
Upper-Limb Coordination: Dropping and Catching a Ball—Both Hands	Basic Concept:<br>Child will drop and catch a ball using both hands as in the BOT-2 exercise.<br>Song Idea:<br>Verse: child can roll the ball back and forth to parent<br>Chorus child must drop and catch the ball using both hands.<br>NMT Technique: PSE	Target # of catches 5/5 attempts

BOT-2 Exercise(s)	NMT Intervention (TIMP, PSE, RAS)	Target Speed/Reps
Upper-Limb Coordination: Dribbling a Ball—Alternating Hands	Basic Concept:<br>Child will alternate dropping and catching the ball using alternating hands like in the BOT-2 exercise.<br>Song idea: As in dropping/catching ball.<br>NMT Technique: PSE	10/10 correct drumming hits
Strength: Knee Push-ups<br>Strength: Full Push-ups	Basic Concept:<br>Child does knee or full push ups (depending on level of skill) for 30 seconds.<br>Song Idea:<br>Therapist can sing child-preferred song during PSE facilitation of movement.<br>NMT Technique: PSE. *Ensure that there are clear force cues particularly in the difficult parts of the push ups.	# of correct push-ups in 30 seconds
Strength: Sit-ups	Basic Concept:<br>Caregiver holds drum at child’s knees. Each sit up, child taps the drum with hands.<br>Song Idea:<br>Therapist can sing child-preferred song during PSE/TIMP facilitation of movement.<br>NMT Technique: TIMP & PSE.	# of drum hits (sit-ups) in 30 seconds.

2 Supplementary Material 2

Fidelity Checklist for Sessions

Participant #:_______ Session Date:_______________

Session Participation:

- Spent at least 75% of session on NMT sensorimotor interventions
  - Comments:

- Internet was consistent throughout session
  - Comments:

- Child participated consistently throughout session
  - Comments:

- Caregiver was fully supportive during session
  - Comments:

Logistics (Check where applicable):

- Participant showed up. _____

- Verbal reminder provided for session time/date for following session. _____

- Checked in with caregiver about how they perceive sessions to be going. _____

- Logged session completion. _____

Other Comments:

3 Supplementary Material 3

Qualitative Responses by Theme and Category

Themes	Categories	Example Quotes
Theme 1: Caregiver Involvement was Necessary and Beneficial	Category 1a (Therapists): Collaborating with Motivated Caregivers was Positive	*"I think what's incredible is how willing these mums and dads were/are at helping to facilitate." (T)*<br>*"Collaborating with the family was really valuable throughout the sessions. They were really able to guide and support the client without me physically being there."*<br>*"Collaborating with mum was really positive and having that support on the other end was very valuable. The participant's mom was highly motivated and listened to instructions given, which made sessions more effective." (T)*<br>*"Having the parents there was helpful and providing unconditional positive regard to the child appeared to keep them engaged." (T)*
	Category 1b (Caregivers): Caregivers Learned through Active Engagement	*"Most beneficial for me: Being an active participant in my son's therapy as opposed to passively watching/learning or not being present at all." (C)*<br>*"We learned how, when, where, and what to give him for his sensory needs, which help him concentrate better and happier with his task." (C)*
Theme 2: Clients Benefited from Sessions	Category 2a (Therapists & Caregivers): Clients made progress and gained new skills	*"Witnessing the progress of the participant and hearing mom's feedback and excitement each week regarding the changes she has been seeing directly related to our sessions." (T)*<br>*"This was a satisfying experience overall as I felt like this client benefitted from these services." (T)*<br>*"We witnessed [our child] progressing well in several areas, making connections to different parts of his body, learning to use left hand motions, listening and following directions, willing to adapt to change in routine, following a tune, singing a song on request and more." (C)*
	Category 2b (Therapists): Music therapy was a new, accessible opportunity for participants.	*"Probably the ability to provide a service that is new to the client/family and a service that they might not necessarily be able to seek out depending on resources and their personal situations." (T)*<br>*"Being able to offer a service that the family had never experienced before and to offer them something that was accessible." (T)*<br>*"Most beneficial for my son: Exposure to a completely different type of therapy than he is used to (i.e. ABA, speech) and using different objects/instruments in a structured way." (C)*

Themes	Categories	Example Quotes
Theme 2: Clients Benefited from Sessions (continued)	Category 2c (Caregivers): Music was engaging for their child, even online	*“[Our child] enjoys music and playing instruments. She enjoyed being part of the study. For my child, in-person class works better in compared to online. But music zoom session something she really look forward to do almost every week.” (C)*<br>*“We have done Speech therapy, ABA therapy and Activity camps through online sessions for [our child]. But this music therapy was totally a different experience for both of us. [Our child] was totally engaging from the 1st session.” (C)*
Theme 3: Engagement on Zoom was Sometimes Limited	Category 3a (Therapists): Visual and Auditory Distractions	*“It was hard to use the metronome effectively in some cases because of varying abilities and sound challenges. I think observing the quality of movements can be difficult at times.” (T)*<br>*“General background noise and distractions in the environment (people talking to each other, talking on cell phones, etc.).” (T)*<br>*“The child was really focused on looking themselves during the session, and that was really challenging in terms of making sure they were attending to the instructions or the task at hand.” (T)*<br>*The most challenging aspect was gauging the actual space and room with the limitation of camera angles.” (T)*
	Category 3b (Caregivers): Difficulty Remaining On-Task	*“Staying on task is bit challenging for my child. Needs redirection.” (C)*<br>*“The most challenging aspect was definitely getting my son to remain engaged. The combination of the medium (virtual) and timing (after school - overtired/overstimulated) made it difficult.” (C)*
